# Human‐mediated introduction of introgressed deer across Wallace's line: Historical biogeography of *Rusa unicolor* and *R. timorensis*

**DOI:** 10.1002/ece3.3754

**Published:** 2017-12-27

**Authors:** Renata F. Martins, Anke Schmidt, Dorina Lenz, Andreas Wilting, Joerns Fickel

**Affiliations:** ^1^ Department of Evolutionary Genetics Leibniz Institute for Zoo and Wildlife Research Berlin Germany; ^2^ Institute for Biochemistry and Biology University of Potsdam Potsdam Germany

**Keywords:** Cervidae, human introduction, hybridization, Phylogeography, Sundaland, Wallace's line

## Abstract

In this study we compared the phylogeographic patterns of two Rusa species, *Rusa unicolor* and *Rusa timorensis*, in order to understand what drove and maintained differentiation between these two geographically and genetically close species and investigated the route of introduction of individuals to the islands outside of the Sunda Shelf. We analyzed full mitogenomes from 56 archival samples from the distribution areas of the two species and 18 microsatellite loci in a subset of 16 individuals to generate the phylogeographic patterns of both species. Bayesian inference with fossil calibration was used to estimate the age of each species and major divergence events. Our results indicated that the split between the two species took place during the Pleistocene, ~1.8 Mya, possibly driven by adaptations of *R. timorensis* to the drier climate found on Java compared to the other islands of Sundaland. Although both markers identified two well‐differentiated clades, there was a largely discrepant pattern between mitochondrial and nuclear markers. While nDNA separated the individuals into the two species, largely in agreement with their museum label, mtDNA revealed that all *R. timorensis* sampled to the east of the Sunda shelf carried haplotypes from *R. unicolor* and one *Rusa unicolor* from South Sumatra carried a *R. timorensis* haplotype. Our results show that hybridization occurred between these two sister species in Sundaland during the Late Pleistocene and resulted in human‐mediated introduction of hybrid descendants in all islands outside Sundaland.

## 1 INTRODUCTION

**Figure 1:**
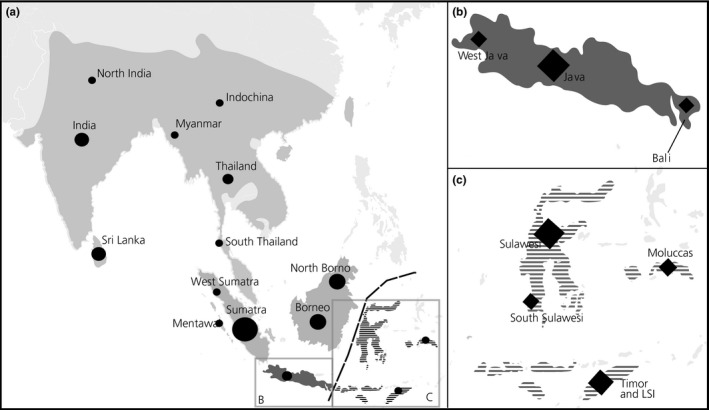
Distribution map of both species and sampling location. Light gray indicates the distribution range of *Rusa unicolor* (a). Dark gray indicates the native distribution of *Rusa timorensis*, (b) and dashed dark gray areas indicate introduction range of *R. timorensis* (C). Filled circles and diamonds indicate *R. unicolor* and *R. timorensis*, respectively, and size is proportional to the number of samples. For detailed information about all samples see Table A1

Biogeographic barriers interrupt migration and reproduction among populations and thus are an important force responsible for driving and maintaining genetic differentiation, potentially leading to speciation. Sundaland, a Southeast Asian biodiversity hotspot, is bordered in the East by one of the best known faunal boundaries—the Wallace line (Bacon, Henderson, Mckenna, Milroy, & Simmons, 2013). This barrier is responsible for a sharp break between faunal compositions of Sunda and Wallacea. At the southern border, the Wallace line runs between the islands of Bali and Lombok, and at the northern edge, it divides fauna and flora of Borneo and the Philippines from that of Sulawesi (Figure 1). Although some species and populations have naturally dispersed across this barrier (squirrel sps.; Mercer and Roth 2003), the presence of the majority of Sundaic mammal species occurring past the Wallace line into the eastern islands of Wallacea is associated with human transportation (Groves, 1983; Heinsohn, 2003; Veron et al., 2014).

Sundaland's dynamic geologic and climatic history, especially during the Plio‐Pleistocene epochs, resulted in sea level changes that repeatedly exposed the continental shelf connecting the major islands of this archipelago (Voris, 2000). It is believed that these available land bridges would allow populations previously isolated on single islands to disperse, creating a large panmictic population within this whole system (Latinne et al., 2015; Demos et al., 2016). However, deep geographical barriers like the Wallace line would remain through even low sea level periods, thus creating patterns of genetic divergence between taxa on both sides of these barriers.

Here, we investigated the phylogeographic patterns of two Rusa species: the sambar, *Rusa unicolor*, and the Javan deer, *Rusa timorensis*. While *R. unicolor* is widespread throughout South and Southeast Asia (from India and Sri Lanka, Southern China and most of Indochina to Borneo and Sumatra, the two largest Sunda Islands), *R. timorensis* has its native range on Java and Bali only (Figure 1). The presence of Javan deer on islands east of the Wallace line (e.g., Lesser Sunda Islands, Sulawesi, and the Moluccas) is described to be the result of prehistoric to historic human‐mediated introductions during the Holocene. These human‐mediated introductions were attributed, for example, to the Austronesian‐speaking peoples’ migrations, ca. 4,000 years ago, responsible as well for the introduction of other deer species (e.g., muntjacs), pigs, macaques, and civets; (Heinsohn, 2003; Groves & Grubb, 2011).

The aim of this study was to compare phylogeographic patterns, genetic diversity, and evolutionary history of these two related species in order to answer the following three questions: (1) In the presence of land bridges connecting islands of the Sunda Shelf, what geographical or climatic barriers were responsible for speciation between the two species and is there evidence of admixture between them? (2) Do populations of the widely distributed *R. unicolor* show signs of genetic structuring corresponding to known geographical barriers? (3) Does *R. timorensis* show a genetic signature of non‐natural dispersal and what is the most likely source population of the introduced populations East of the Wallace line?

## 2 MATERIALS AND METHODS

### 2.1 Sampling and DNA extraction

We sampled 110 individuals labeled as *Rusa unicolor* (RUN) and *Rusa timorensis* (RTI) from European museums, aged between 180 and 61 years old. We collected either turbinal bones from the nasal cavity, skin, dry tissue from skeletons, and antler drills exclusively from individuals with known locality. All molecular work, including DNA extraction and sequencing library preparation, was conducted in a laboratory dedicated to work with archival samples to reduce the risk of contamination. DNA extraction followed the DNeasy Tissue and Blood kit protocol (Qiagen, Hilden, Germany), with overnight digestion of samples in Lysis buffer and Proteinase K at 56°C and a pre‐elution incubation for 20 min at 37°C.

### 2.2 Mitochondrial genome

All extractions, including negative controls, were built into individual sequencing libraries with single 8‐nt indexes (Fortes & Paijmans, 2015), which were then sequenced on an Illumina MiSeq to assess sample DNA quantity and quality (150 cycles v3 kit, Illumina, CA, USA), as described below. Samples with low‐quality DNA were consequently enriched for their mitochondrial DNA, using an in‐solution target hybridization capture technique (Maricic, Whitten, & Pääbo, 2010). Baits for hybridization were obtained by amplifying three overlapping mitochondrial fragments from one fresh tissue sample of *R. unicolor* (from the IZW archive) which were consequently prepared into capture baits (Maricic et al., 2010; primers and PCR conditions as described in Martins et al., 2017). After hybridization capture, libraries were amplified for no more than 18 cycles and sequenced again on the Illumina MiSeq platform.

### 2.3 Bioinformatics

Sequencing reads were first demultiplexed into respective samples with BCL2FASTQ v2.17 (Illumina, CA, USA). CUTADAPT v1.3 (Martin, 2011) was used to find and remove adapter sequences from the sequenced reads. Adapter‐clipped reads were then quality trimmed through a sliding window approach of 10 bp for a phred score of at least Q20. Finally, reads shorter than 20 bp were removed from further analyses. Mapping of quality‐filtered reads was carried out in two phases: A first mapping run was performed with BWA v.0.7.10 (Li & Durbin, 2009), using a genome reference from *R. unicolor dejeani* (NCBI accession no. NC_031835). Clonal reads were removed from the mapped reads using MARKDUPLICATES v1.106 (http://picard.sourceforge.net/picard-tools). SAMTOOLS MPILEUP v1.1 and BCFTOOLS v1.2 (http://github.com/samtools/bcftools) were used for variant calling (SNPs and InDels). A consensus sequence was then generated for each sample using a threshold of minimum 3× coverage and majority rule (>50%) for base calling. The second mapping step used the newly generated consensus sequence as reference for each sample, in order to increase mapping quality and base coverage. BWA and MARKDUPLICATES were used as before, but GATK v1.6 (McKenna et al., 2010) was applied for variant calling of the final consensus sequence. Positions with coverage lower than 5× were N‐masked, as were ambiguous heterozygous positions. A final quality filtering step was performed to remove all samples with less than 80% of their mitogenome covered at least with 5× depth. Due to the limitations of the sequencing method (reads no longer than 75 bp), we were not able to clearly resolve the repeat region of the d‐loop. Therefore, we trimmed all sequences, by removing 460 bp of the d‐loop region. Mitogenomes obtained were deposited in Genbank (for accession numbers see Table A1).

### 2.4 Microsatellite DNA

Microsatellite genotyping was achieved by amplification of 18 loci on all 56 samples for which mitochondrial DNA was obtained as described above (microsatellite loci and references in Table A2). All samples were amplified through PCR with the Type‐it Microsatellite PCR kit (Qiagen, Hilden, Germany), with 1 μmol/L of each primer. Annealing temperatures followed a gradient from 63°C to 55°C in 2°C steps and final amplification occurred for 40 cycles at 55°C. Allele sizes were determined on an ABI3130*xl* Genetic Analyser using GeneScan^™^ 500 ROX (both Thermo Fischer Scientific, Darmstadt, Germany) as internal size standard. Alleles were scored with the software GeneMapper v.4.0 (Applied Biosystems, Germany).

### 2.5 Genetic diversity, phylogeography, and differentiation times

All mitochondrial sequences obtained were aligned using the auto setting as implemented in MAFFT v7.245 (Katoh & Standley, 2013). The relationship among all haplotypes was reconstructed by a median‐joining (MJ) network using the software NETWORK v. 4.6.1.4 (Bandelt, Forster, & Röhl, 1999). Haplotypes were generated by removing noninformative sites and positions with gaps or missing data. Haplotypic and nucleotide diversities for the full dataset and for each species were assessed with the software DNASP v.5.10 (Librado & Rozas, 2009). We estimated genetic differentiation through F_ST_ as implemented in ARLEQUIN v.3.5.12 (Excoffier, Laval, & Schneider, 2005). For this analysis, we created two datasets: (1) two populations corresponding to species as determined by the museum identification and (2) populations corresponding to major haplotype clades.

The best fitting substitution model for the full mitogenome dataset (GTR + G + I) was obtained by the hierarchical likelihood ratio test as implemented in JMODELTEST v2.1.7 (Darriba, Taboada, Doallo, & Posada, 2015). We reconstructed phylogenetic relationships through maximum likelihood (ML) with RAXML GUI v1.5 (Silvestro & Michalak, 2012) and Bayesian inference (BI) as implemented in MRBAYES v3.2.6 (Ronquist & Huelsenbeck, 2003), applying the determined substitution model. Both approaches were congruent with the haplotypic network and with each other. We dated the BI tree based on fossil information for the Cervidae stem of the Arctiodactyl family (18.4 Mya; Bibi, 2013). For that, we first determined divergence dates for a dataset containing *Bos javanicus* as an outgroup (JN632606.1), *Muntiacus reevesi* (AF527537.1), *Axis axis* (NC_020680.1) and *A. porcinus* (JN632600.1), *D. dama* (NC_020700.1), *Cervus nippon* (JN389444.1) and *C. elaphus* (AB245427.2), and the two Rusa species investigated here, using the software BEAST (Drummond et al. 2012). We ran two MCMC chains with 50 million, with a lognormal uncorrelated clock and a Yule speciation tree as further estimation priors. We used the calibrated time of the split between Cervus and Rusa (2.1 My; Highest Posterior Density [HPD] = 1–2.8 My) to be set as a prior for the root height of the gene tree of the Rusa samples obtained in this study, as before. Trace results were analyzed with TRACER v.1.6 (implemented in BEAST v.1.8), for parameter convergence and ESS values above 200. TREEANNOTATOR v.1.8.1 (BEAST software package) was used to annotate all trees, after a burn‐in of 10% of the trees. All topologies were visualized and edited with the software FIGTREE v.1.4.2 (http://tree.bio.ed.ac.uk/software/figtree/).

### 2.6 Population structure analyses

Analyses of microsatellite data proceeded by removing all individuals with missing data at more than two loci. We estimated the probability for the presence of null alleles on our dataset with the software FREENA (Chapuis & Estoup, 2007). Tests for the presence of linkage disequilibria and an exact test for deviations from Hardy–Weinberg Equilibrium (HWE) were performed with ARLEQUIN, applying the Bonferroni correction for multiple tests. Levels of observed (*H*_O_) and expected (*H*_E_) heterozygosity and inbreeding coefficient (*F*_IS_) were calculated in GENETIX v.4.05.2 (Belkhir, Borsa, Chikki, Raufaste, & Bonhomme, 1996).

A Bayesian approach was used to test for population structure with the software STRUCTURE 2.3.4 (Pritchard, Stephens, & Donnelly, 2000). The λ value was estimated by running a prospective run of K=1 with 10 iterations and a burn‐in of 10% after 15 × 10^4^ generations. A second MCMC simulation was run for 20×10^4^ generations, with a 10% burn‐in. The likelihoods were estimated for K values from 1 to 6, because 6 was higher than the maximum number of mitochondrial clades obtained in our analyses. The admixture model was applied with correlated allele frequencies and λ = 3. STRUCTURE HARVESTER v.0.6.94 (http://taylor0.biology.ucla.edu/structureHarvester/; Earl & von Holdt, 2012) was used to estimate the most likely number of K using the ∆K method (Evanno, Regnaut, & Goudet, 2005). Population differentiation was calculated with the software ARLEQUIN by estimating *F*_ST_ both among the clusters identified by ∆K and by species.

## 3 RESULTS

### 3.1 Mitochondrial genome analyses

The final dataset consisted of 56 individuals for which a mitogenome of 16,064 bp was obtained. Of these, 23 samples were labeled in the museum collections as *R. timorensis* and 33 individuals were labeled as *R. unicolor* (Table A1). The origin of three specimens labeled as *R. unicolor* (RUN37 Moluccas, RUN39 Timor, and RUN61 Java) suggests that these specimens are actually Javan deer, *R. timorensis*. All other museum labels matched the geographical distribution ranges of the two species. The 56 deer shared 46 haplotypes with an overall haplotype diversity of H = 0.983 (*SD* 0.010) and a very low nucleotide diversity of π = 0.00941 (*SD* 0.00118). Within individuals labeled as RTI, we found 18 haplotypes with H = 0.972 (*SD* 0.026) and π = 0.0085 (*SD* 0.0024). Within RUN‐labeled individuals, there were 28 unique haplotypes, and one haplotype was shared due to admixture with RTI (H_3). Overall, the 29 haplotypes had H = 0.991 (*SD* 0.011) and π = 0.011 (*SD* 0.0012).

**Figure 2:**
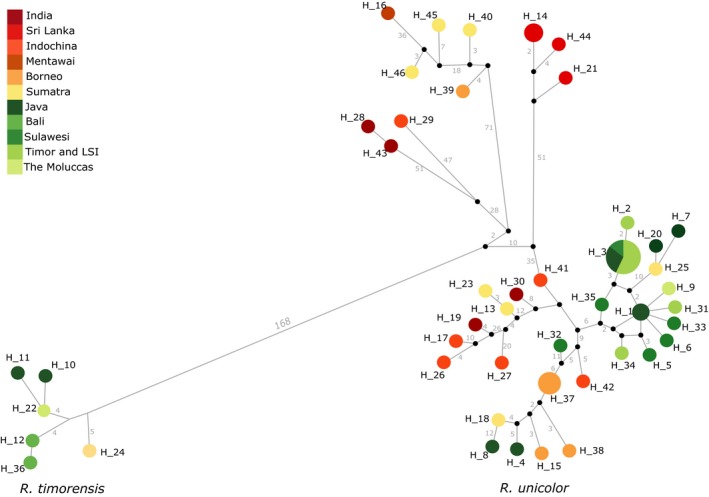
Haplotypic network for all 46 haplotypes shared among the two species. Circle size is in accordance with frequency and color represents sampling location. Small black circles represent median vectors. All branches represent one mutation step, except when indicated otherwise by numbers on branches. Two major clades were recovered and are indicated by the species names. Haplotypes are described in Table A1

**Figure 3:**
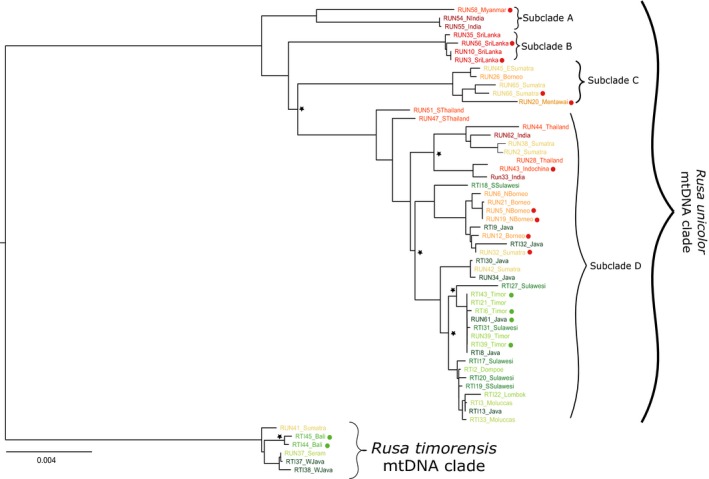
Mitogenome maximum likelihood tree of both species. Colors on tips represent sampling location (as in Figure 2) and stars represent split events with bootstrap values/Bayesian posterior probabilities lower than 90/0.95 (but bigger than 50/0.5). Red and green dots represent samples for which we obtained nDNA; red dot: assigned to the *Rusa unicolor* genotypic cluster and green dot: assigned to the *Rusa timorensis* genotypic cluster. Major mtDNA clades and subclades are labeled with curved brackets. Scale bar indicates number of substitutions per position

The full mtDNA haplotype network separated two major clades by a minimum of 168 mutational steps (Figure 2). The smaller clade comprised six individuals from Java, Bali, Moluccas, and South Sumatra (Figure 2) of which four had been labeled in the museum collections as *R. timorensis* (Javan deer, RTI) and two as *R. unicolor* (sambar, RUN; RUN41: South Sumatra; RUN37: Moluccas, see above). The second major clade comprised all other samples, including samples labeled as RTI from Java and from the introduction range of Javan deer (Lesser Sunda Islands, Sulawesi, and the Moluccas). Both ML and BI tree topologies were concordant with the overall pattern recovered by the haplotypic network, also revealing the existence of two well‐differentiated clades (Figure 3). Generally, genetic diversity of haplotypes showed geographical structure only in some parts of the tree (subclades A, B, and C). Samples from the Moluccas, Sulawesi, and Timor (outside Sundaland) were present in more than one branch of the phylogenetic tree (subclade D).

**Figure 4:**
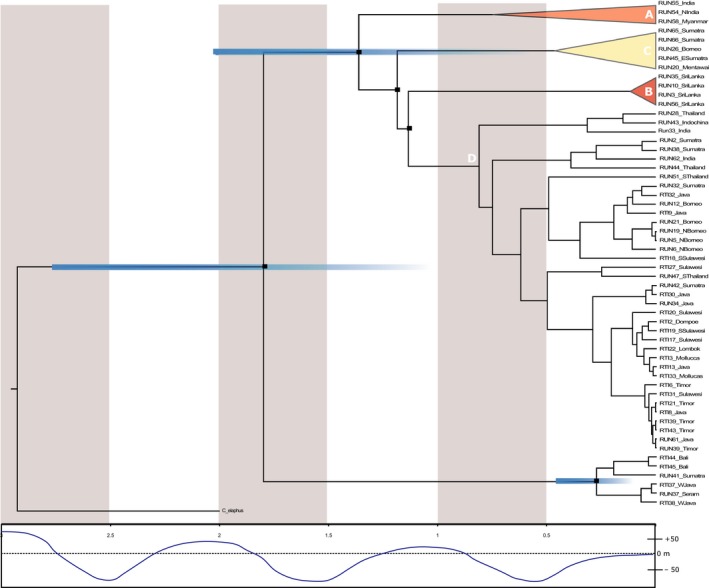
Mitochondrial DNA dated tree according to BEAST analyses, from 3 Mya to present. Blue bars represent associated deviations for the most important splits. A time scale in millions of years and a rough estimate of sea level changes through time (adapted from Patou et al. 2010) are presented below

The resulting node ages for the estimated age of Cervidae species were similar to those reported in other studies (Table 1). Using those, we then estimated the timing of divergence between the two main clades to have started in the early Pleistocene, about 1.8 million years ago (Mya) (HPD = 0.95–3.1). The position of all clades was similar to the topology recovered by ML, with the exception of the Sri Lankan clade position (clade B, Figures 3 and 4), which diverged more recently in the BI tree. This subclade was also accompanied by low Bayesian Posterior Probability values (BPP = 0.34). Nevertheless, our divergence estimates indicated that subclade A diverged first within the RUN mitogenome clade about 1.4 Mya (HPD = 0.7–2.3); subclade C diverged 1.185 Mya (HPD = 0.6–2) and subclade D at around 1.13 Mya (HPD = 0.6–2) (Figure 4). *F*_ST_ showed significant population differentiation only when populations were based on mtDNA clade assignment, but not when they were based on species assignment from the museum collections (Table 2).

**Table 1:** Calibrated divergence dates estimated for the Cervidae tree

Split	Age			Other studies
	Median	Minimum	Maximum	
Bovidae/Cervidae	16.8	10.7	23.3	**18.4** (Bibi, 2013)
Muntiacus/Cervus	7.2	4.1	10.2	7.5 (Martins et al. 2017)
Axis/Cervus/Dama	4.6	2.6	5.4	**6** (Di Stefanio & Petronio, 2002)
Cervus/Rusa	2.1	1	2.8	**3.4** (Pitra et al. 2004)
*R. unicolor*/*timorensis*	1.4	0.7	2.2	–

Ages (in million years [My]) represent the median obtained for each of the described split. Values in bold represent fossil‐based calibrations.

**Table 2:** Population differentiation estimates (*F*_ST_) according to marker and grouping

	*F*_ST_	*p*‐value
mtDNA		
Museum ID	0.085	>.001
Results (2 clades)	0.75	**<.001**
nDNA		
Museum ID	0.12	**<.001**
Results (K = 2)	0.14	**<.001**

Estimations were performed both for the mtDNA and nDNA. Populations were generated by either museum ID (both for mtDNA and nDNA) and by assignment of individuals to one of the two major clades (mtDNA) or to one of the genotypic clusters (nDNA). Statistically significant comparisons are indicated by bold *p*‐values.

### 3.2 Microsatellite analyses

Of all archival samples for which mitogenomes were obtained, 16 individuals (~29%) could be successfully genotyped at 18 loci. These individuals were distributed relatively well across the clades of the mitogenome tree (Figure 3). Linkage disequilibria were found at 10% of all pairwise loci combinations, yet without any consistency, and percentage of null alleles was 0.18. Therefore, we retained all loci for further analyses. We detected significant deviations from HHWE, which indicated probable population structure within our dataset. Expected and observed heterozygosities at each locus ranged from 0.23 (locus Mu_4D) to 0.92 (locus Mu_1_51) and from 0 (locus Mu_4D) to 0.61 (locus Roe09), respectively (Table A2). Number of alleles varied among loci, with the highest number found at loci Mu_1_51 and NVHRT48 (16 alleles) and the lowest found at locus Mu_4D with only two alleles (Table A2).

**Figure 5:**
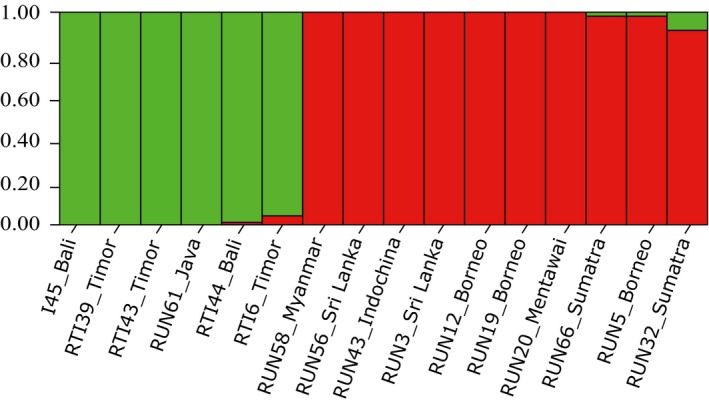
Genotyping results from 16 individuals genotyped for 18 loci, showing a structure plot for K = 2, with *R. timorensis* samples in green and *R. unicolor* individuals in red. Each column represents a single individual, as identified below

According to the ∆K approach, the most likely number of genotypic nDNA clusters was K = 2 (Figure 5). These two main clusters corresponded well to the two species, sambar (*R. unicolor*, green cluster) and Javan deer (*R. timorensis*, red cluster). Population differentiation (*F*_ST_) was always significant between the two species, independent of the grouping method (museum assignments or STRUCTURE analyses, Table 2).

## 4 DISCUSSION

The mitochondrial genomes and the nDNA loci of sambar and Javan deer investigated here revealed an intriguing and surprising pattern of genetic diversity and population differentiation between the two species. Although monophyly of *R. timorensis* and *R. unicolor* remain undisputed, our results point to a more complex history of hybridization between species and multiple human‐mediated introductions outside the Sunda Shelf.

The presence of two divergent matrilineages clearly indicates molecular differentiation between two groups of Rusa deer, which we interpret as the historical cladogenesis of both *R. timorensis* and *R. unicolor*. Our fossil‐calibrated estimates are corroborated by recent studies (Bibi, 2013; Escobedo‐Morales, Mandujano, Eguiarte, Rodriguez‐Rodriguez, & Maldonado, 2016; Table 1) and are in accordance with the dates suggested by other authors for the age of the genus Rusa (e.g., 2–2.5 Mya; Di Stefano & Petronio, 2002). The separation of the two deer species investigated here had been challenged in the past (mentioned in Van Bemmel, 1949), yet subsequent studies found robust support for their distinctiveness (morphological: van Bemmel, 1994; Meijaard & Groves, 2004; and molecular: Emerson & Tate, 1993; Pitra, Fickel, Meijaard, & Groves, 2004). Our comprehensive molecular study corroborated these findings, but also provides evidence for a much more complex evolutionary history of the Rusa deer. It has been proposed that Rusa‐like deer have appeared in Northern India, around 2.5 Mya, where they adapted to dense forest habitats with some open grass vegetation (Geist, 1998). However, during the Pleistocene, subtropical forest shifted southwards, completely disappearing from China (Meijaard & Groves, 2004). This would have also shifted the distribution area of subtropical forest‐adapted Rusa (or Rusa‐like) species southwards too. When low sea level allowed, Rusa deer could have reached Sundaic islands, including Java. Sea level remained low until 1.4 Mya, maintaining connections between landmasses through the emerged continental shelf (Van Den Bergh, De Vos, & Sondaar, 2001). By 1 Mya, sea level had risen again and had reached a highstand at +5 m compared to present day (Zazo, 1999), thereby interrupting land bridges between islands. At this time, Rusa populations of Java and Sumatra (clade D) would have become isolated and habitat availability for forest‐dependent species would have been reduced.

Our data indicate that there was also a second wave of colonization to Sundaic islands by *Rusa unicolor*, likely from Thailand (Mainland). This second wave would have likely occurred during the Late Pleistocene, with drops in sea levels and once again cooler and drier climates. This southward expansion brought previously isolated *Rusa unicolor* in contact with *Rusa timorensis* from Java, facilitated by the presence of the emerged Sunda Strait, a strait that submerged just ~10 kya (Sathiamurthy & Voris, 2006). This fact raises the obvious question of what then maintained differentiation between the two species and/or restricted hybridization to a small secondary contact region. We hypothesize that the different ecological niches on Java and Sumatra might have had a central role. Speciation may have resulted from the ecological adaptation of Javan deer *Rusa timorensis* to the prevailing vegetation type on Java, separating it from its sister species, the subtropical forest‐adapted sambar *Rusa unicolor*. Java and Bali, although part of Sundaland, had (and still have) different climatic conditions than Sumatra and Borneo and thus allowed differentiation between species based on evolved ecological adaptations (Leonard et al., 2015). The climate on Java is characterized by a West–East gradient, a transition from a slightly seasonal climate in the West to a strongly seasonal one in the East. Central and East Java are characterized by drier, cooler climate (climate‐data.org), and the vegetation has more grass areas than on the surrounding islands (Heany, 1991; Mishra, Gaillard, Hertler, Moigne, & Simanjuntak, 2010). Therefore, it is likely that *R. timorensis*, being better adapted to drier climate, would have crossed the dry central Sundaland during Pleistocene glacials to colonize east Java (Sheldon, Lim, & Moyle, 2015), where it stayed isolated from its sister species. After this initial separation, we find evidence of range expansion, likely during consequent drops in sea levels, demonstrated by the introgression in Java and possibly South Sumatra.

One sambar individual from South Sumatra was found to carry RTI mtDNA. This indicates the possibility that individuals of *R. timorensis* also migrated to at least South Sumatra, where they hybridized with *R. unicolor*. Such a range expansion would have been enabled by the drier and cooler climates and the emerged land corridor between Sumatra and Java during the Late Pleistocene, as at the time of LGM, West Java presented drier and cooler climates in the lowlands (Sun, Li, Luo, & Chen, 2000). Because we did not obtain the genotype of this sample, more intensive sampling of South Sumatran populations would be required to conclude that these results reflect evidence of reciprocal hybridization between two sister species of deer. On the other hand, we found a strong evidence that the sambar, likely during wetter interglacial periods, expanded its range from Sumatra at least to western Java where it hybridized with the Javan deer. Those introgressed Javan deer most likely constituted the source population for the introductions east of the Wallace line.

### 4.1 Phylogeography and taxonomy of *Rusa timorensis*

Within *R. timorensis*, individuals from Bali and West Java showed genetic divergence at mtDNA. This substructure could indicate limited gene flow during parts of the Late Pleistocene, which might corroborate the classification of Bali populations as *R. t. renschi*, with genetic isolation most likely being the result of a “small population effect” and a limited/interrupted gene flow to Javan populations. However, because we only had two samples from Bali and no samples from East Java, our assessment has to be viewed cautiously. It does however indicate the especially urgent need to assess if hybridization occurred as well in these populations, through more extensive sampling and inclusion of nuclear markers.

The mtDNA of RTI‐labeled samples from islands beyond the Wallace line clustered with *R. unicolor* mtDNA but did not show any clear geographical distribution pattern. These RTI hybrids shared haplotypes with RUN‐labeled samples from Sumatra and Borneo (Figures 2 and 3). Genetic distances among RTI hybrid haplotypes and to other RUN haplotypes were very low, indicating a recent, thus human‐mediated introduction to these Wallacea islands. Quite recently, it had been suggested to split *R. timorensis* into seven subspecies according to their occurrence on islands within and outside of the Sunda shelf (Mattioli, 2011; Hedges, Duckworth, Timmins, Semiadi, & Dryden, 2015). However, our data do not support such a suggestion, as all samples from the introduction range shared haplotypes, indicating a lack of differentiation among individuals from these Wallacean islands. Furthermore, the few samples from Java and Timor that could be genotyped showed genetic similarity, again indicating the lack of differentiation.

### 4.2 Phylogeography and taxonomy of *Rusa unicolor*

Sambar is currently subdivided into five subspecies: *R. u. unicolor* (India, Nepal, Bangladesh, and Sri Lanka), *R. u. brookei* (Borneo), *R. u. cambojensis* (mainland Southeast Asia, from South China/Hainan and Myanmar to Peninsula Malaysia), *R. u. equine* (Sumatra and Mentawai), and *R. u. swinhoei* (Taiwan; Mattioli, 2011). The mitogenome structure recovered here, however, did not support any of the described subspecies, as it indicated gene flow between all populations. Especially among populations of Sundaic islands, we found a lack of genetic structure that would correspond to isolated islands, evidenced by the presence of individuals distributed throughout the tree topologies and haplotypic inferences.

Among populations of sambar, we found evidence of at least three deep split (>1 My) subclades which were not in accordance with the current subspecies assignment. Subclade A comprised haplotypes from Myanmar and India, with an age of about 1.36 My; the second clade included Sundaic populations from Sumatra, Mentawai, and Borneo (clade C) and was dated to be ~1.18 My old; and the third one included all haplotypes from Sri Lanka (clade B) and split from the remaining populations ~1.13 Mya. Subclade D encompassed all remaining individuals of sambar, both from Mainland South and Southeast Asia and the Sundaic islands. Despite the ancient split of clade A further sampling of mainland southeast Asia, particular India, Myanmar, and Bangladesh is needed to reveal whether clade A is indeed geographically separated from the other mainland Asian populations, particular those represented in subclade D. Thus, albeit historically clade A and D were isolated, our data indicate recent gene flow between the two clades, and thus, it remains uncertain whether our data would support a subspecific status of individuals from clade A. Very similarly, Sumatran individuals were present in the distinct mitochondrial clades C and D, and thus, our data did not support separating these clades in distinct taxonomic units.

These subclades could then rather represent centers of sambar distribution, which would have remained in place during times of warmer and wetter conditions, which contracted sambar populations to subtropical refugia. From these centers, we observed waves of expansion. The branching order of these three old subclades indicated colonization from northern Indochina southwards to Sri Lanka and to the Sunda Shelf, respectively. The “younger” individuals within subclade D from India, as well as from Sumatra and Borneo, appear then to be descendants from a second/third natural dispersion wave (possibly from Thailand) during glacial periods of the Pleistocene, when low sea level again exposed the shallow Sunda shelf connecting all major islands (Voris, 2000; Bird, Taylor, & Hunt, 2005). During glacial periods, climate was drier and cooler in tropical regions (Gorog, Sinaga, & Engstrom, 2004). However, species that retained a broad ecological niche such as *Rusa unicolor* would have been able to utilize the newly emerged habitats Such a scenario would likely cause the haplotypic distribution pattern we observed here. During glacial periods, Sundaland was also connected to Southeast Asian mainland, allowing secondary admixture between formerly separated populations, thus generating the patterns we observe between haplotypes from Thailand, India, and Sundaland.

In contrast, the distinct Sri Lankan clade B provides additional evidence for the recognition of Sri Lankan sambar as being distinct. This support comes both from morphological assessments (Groves & Grubb, 2011) and karyotype differences (2n = 56 in Sri Lankan sambar vs. 2n = 58 in Indian and 2n = 62 in Chinese and Malaysian sambar; Leslie, 2011). Sri Lankan populations are often more genetically related to the Western Ghats than to other Indian regions. Very recently, a 40 bp insertion was detected in the control region of the mitochondrial DNA in samples from the Western Ghats (Gupta, Kumar, Gaur, & Hussain, 2015), whose presence we, however, were unable to verify due to method limitations.

Thus, further studies on the mainland populations that could confirm the presence of old splits within *R. unicolor* are of urgency. Increased sampling and, especially, the inclusion of nuclear markers could confirm the extent of gene flow between these populations or, conversely, true genetic divergence between the subclades recovered here. If confirmed, these populations might represent subspecies of sambar occurring in highly disturbed regions of Mainland Southeast Asia.

### 4.3 Introductions past the Wallace line

It is generally accepted that the presence of Rusa deer on islands beyond Sundaland (excluding Philippines) was the result of human interference (Long, 2003; Groves & Grubb, 2011; Hedges et al., 2015). However, until now, these individuals were assumed to have been pure *R. timorensis*, collected, and transported for venison and as game species by humans from the islands of Java and Bali during the Holocene (Heinsohn, 2003). While the nDNA data clearly separated the two species—being highly concordant with their description from the museum collections—the mitochondrial genomes point to a more surprising pattern of past Pleistocene hybridizations. Human‐mediated introductions of these deer occurred ca. 3,000 years ago (Heinsohn, 2003), and, to our knowledge, these results could therefore constitute evidence for the earliest transport of introgressed deer.

All mtDNA haplotypes of samples labeled in the museum collections as *R. timorensis* and sampled from Wallacean islands (Sulawesi, Lesser Sunda Islands and The Moluccas) were of *R. unicolor* origin, rendering these individuals hybrid descendants. The most parsimonious explanation for the molecular patterns obtained in this study is that hybridization occurred on Java (center of star‐like pattern in the haplotypic network), with natural dispersion of female sambar. These immigrated individuals were likely from Sumatra and/or the Thai‐Malay Peninsula, as indicated by the basal position of the two Southern Thailand individuals (RUN51 and RUN57), and they would have used the connecting land bridges. After the introgression of Sambar haplotypes into Javan populations, humans would then have transported the introgressed descendants of Pleistocene hybrids from Java to Timor, the Moluccas and Sulawesi. Despite evidence for multiple independent introductions (Section 2.1), almost all introduced individuals carried sambar haplotypes (except RUN37 from Seram, the Moluccas). This indicates that either humans selected for individuals to be introduced (e.g., carrying a particular trait only found in introgressed Javan deer); that the introgressed individuals had a higher surviving probability after their introduction; or that most introductions occurred from a single region (e.g., West Java) where RUN haplotypes got fixed, possible through mitochondrial capture of sambar haplotypes.

Introduction of Javan deer to Timor seems to have occurred only once and, presumably, with very few founders because of the lack of mtDNA diversity found among all individuals. In fact, populations recently introduced to Australia and New Caledonia from a low, known number of individuals from Timor, have been shown to have very low genomic diversity, which would be the expected result after an introduction of individuals that had come from an already genetically impoverished population (Webley, Zenger, English, & Cooper, 2004; de Garine‐Wichatitsky et al., 2009). Although we obtained only very few samples from the Moluccas and other Lesser Sunda Islands (Dompoe and Lombok), they did not share haplotypes, indicating either multiple introductions or a higher number of founders. Sulawesi had by far the most genetically diverse Javan deer population of all the Wallacean islands. Its haplotypes were present in almost all younger clades of the mtDNA tree. This pattern indicated that Rusa deer reached Sulawesi multiple times. One sample (RTI18) was closer related to Bornean populations than the other haplotypes from Sulawesi. Although natural dispersal from Borneo to Sulawesi over the Makassar Strait is conceivable, it is highly unlikely as the last possible connection between these two land masses was during the late Pliocene/early Pleistocene ~2.5 Mya, a date that by far predates the emergence of this mtDNA lineage. The most likely scenario is a human‐mediated introduction of Bornean sambar to Sulawesi where it hybridized with the introduced Javan deer. If true, this represents a second hybridization event on Sulawesi (compared to the Late Pleistocene hybridization in Java and potentially South Sumatra) and further studies on Sulawesi Javan deer would be required to test this hypothesis. The remaining haplotypes from Sulawesi individuals were closely related to the haplotypes from individuals introduced to the Moluccas and Timor Islands, indicating either that all of them have been introduced in one wave or at least from a similar source population from Java.

This is the first report of historical hybridizations between sambar (*R. unicolor*) and Javan deer (*R. timorensis*). Occurrence of such hybridization had been documented before, namely between *R. timorensis* individuals introduced to Borneo with the local Bornean *R. unicolor* (West Kalimantan, now possibly extinct, Hedges et al., 2015) and attained through husbandry before (Leslie, 2011). Hybridizations with fertile offspring have also been reported to occur between other deer species, among others between sambar and red deer *Cervus elaphus* (Muir et al.,^1997^) and red deer and Sika *Cervus nippon* (Smith, Carden, Coad, Birkitt, & Pemberton, 2014).

This fact has potentially important conservation implications for the two Rusa species analyzed in this study. Despite being one of the most widespread deer species in southern Asia, *R. unicolor* is today no longer abundant throughout most of its native range (Timmins et al., 2015). Likewise, *R. timorensis* is currently considered a pest species in areas where it has recently been introduced (e.g., Australia) but has, however, decreased largely in population numbers in native and historical introduction regions (Java, Lesser Sunda Islands, Sulawesi, and the Moluccas). Both Rusa species studied here are now considered vulnerable by the IUCN/Red List of Threatened Species (Hedges et al., 2015; Timmins et al., 2015). Therefore, genetic monitoring of individuals, both at mtDNA and particularly also nuclear genomes, is necessary to assess whether pure RTI and RUN individuals are being introduced (or reproductively assisted in their native ranges) and not introgressed individuals. Moreover, more intensive and extensive sampling of *R. timorensis* on their native range is necessary to discern whether pure RTI populations still remain in Java and Bali or whether they are composed in their majority by hybrid individuals.

## 5 CONCLUSION

In addition to representing the first comprehensive phylogeographical study on *R. unicolor* and *R. timorensis*, this study revealed surprising evolutionary histories of these two sister species. Answering the questions poised before, we hypothesized that while climate adaptations were likely responsible for maintaining species monophyly, Pleistocene climate changes were responsible for secondary contact and consequent hybridization between sambar and Javan deer. We recovered a pattern of (possibly reciprocal) introgressions between the two species, facilitated by the presence of land corridors during periods of low sea levels in Sundaland. The introgressed populations of Javan deer on Java were then the source of all human‐mediated introduction waves to the islands east of the Wallace line, as we found that all *R. timorensis* individuals carried *R. unicolor* haplotypes. Additionally, these dramatic climate changes were also likely responsible for the divergence of populations within *R. unicolor*.

## CONFLICT OF INTEREST

None declared.

## AUTHOR CONTRIBUTIONS

RFM, AW, and JF designed the study; RFM and AS performed the laboratory procedures; RFM and DL performed the bioinformatic analyses; RFM, AW, and JF wrote the manuscript. All authors contributed equally for the final version of this manuscript.

## Appendix 1

### 1.1

**Table A1:** Complete dataset details

Species	Sample ID	Origin	MtDNA haplotype	Accession Number	nDNA assignment	Museum Collection	Museum ID		Curator
*Rusa unicolor*	RUN2	Sumatra	H_13	MF176993		Berlin NHM	75150	F. Mayer	
*Rusa unicolor*	RUN3	Sri Lanka	H_14	MF177018	RUN	Berlin NHM	75133	F. Mayer	
*Rusa unicolor*	RUN5	North Borneo	H_37	MF177019	RUN	Berlin NHM	11259	F. Mayer	
*Rusa unicolor*	RUN6	North Borneo	H_38	MF177020		Berlin NHM	11261	F. Mayer	
*Rusa unicolor*	RUN10	Sri Lanka	H_14	MF176994		Berlin NHM	12368	F. Mayer	
*Rusa unicolor*	RUN12	Sarawak, Borneo	H_15	MF176995	RUN	Berlin NHM	14702	F. Mayer	
*Rusa unicolor*	RUN19	North Borneo	H_37	MF177021	RUN	Berlin NHM	103292	F. Mayer	
*Rusa unicolor*	RUN20	Mentawai	H_16	MF176996	RUN	Naturalis	139–50	P. Kamminga	
*Rusa unicolor*	RUN21	Borneo	H_37	MF177022		Stuttgart NHM	16900	S. Merker	
*Rusa unicolor*	RUN26	Borneo	H_39	MF177023		Stuttgart NHM	15900	S. Merker	
*Rusa unicolor*	RUN28	Thailand	H_17	MF176997		Bonn NHM	ZFMK_MAM_2013.618	R. Hutterer	
*Rusa unicolor*	RUN32	Sumatra	H_18	MF176998	RUN	Vienna NHM	7333	F. Zachos	
*Rusa unicolor*	RUN33	India	H_19	MF176999		Vienna NHM	40867	F. Zachos	
*Rusa unicolor*	RUN34	Java	H_20	MF177000		Senckenberg Frankfurt	15937	I. Ruf	
*Rusa unicolor*	RUN35	Sri Lanka	H_21	MF177001		Paris NHM	1877‐10	G. Veron	
*Rusa unicolor*	RUN37	Burum, Moluccas	H_22	MF279250		Munich NHM	1906/3021	M. Hiermeyer	
*Rusa unicolor*	RUN38	Sumatra	H_23	MF177002		Munich NHM	1905/46	M. Hiermeyer	
*Rusa unicolor*	RUN39	Timor	H_3	MF177003		Munich NHM	1911/2147	M. Hiermeyer	
*Rusa unicolor*	RUN41	South Sumatra	H_24	MF279251		Munich NHM	1908/465	M. Hiermeyer	
*Rusa unicolor*	RUN42	Sumatra	H_25	MF177004		Munich NHM	1908/466	M. Hiermeyer	
*Rusa unicolor*	RUN43	Indochina	H_26	MF177005	RUN	Munich NHM	1962/235	M. Hiermeyer	
*Rusa unicolor*	RUN44	South Thailand	H_27	MF177006		Copenhagen NHM	M1594	D.K. Johansson	
*Rusa unicolor*	RUN45	East Sumatra	H_40	MF177024		Copenhagen NHM	M1650	D.K. Johansson	
*Rusa unicolor*	RUN47	South Thailand	H_41	MF177025		Copenhagen NHM	M1593	D.K. Johansson	
*Rusa unicolor*	RUN51	South Thailand	H_42	MF177026		Copenhagen NHM	M1583	D.K. Johansson	
*Rusa unicolor*	RUN54	Bhutan Dooars, India	H_43	MF177027		Copenhagen NHM	M534	D.K. Johansson	
*Rusa unicolor*	RUN55	Bhutan Dooars, India	H_28	MF177007		Copenhagen NHM	M533	D.K. Johansson	
*Rusa unicolor*	RUN56	Sri Lanka	H_44	MF177028	RUN	Copenhagen NHM	M1236	D.K. Johansson	
*Rusa unicolor*	RUN58	South Myanmar	H_29	MF177008	RUN	Naturalis	RMNH.MAM.693.a	P. Kamminga	
*Rusa unicolor*	RUN61	Central Java	H_3	MF177009	RTI	Naturalis	RMNH.MAM.33832	P. Kamminga	
*Rusa unicolor*	RUN62	Bengala, India	H_30	MF177010		Naturalis	RMNH.MAM.51460	P. Kamminga	
*Rusa unicolor*	RUN65	South Sumatra	H_45	MF177029		Naturalis	RMNH.MAM.51453	P. Kamminga	
*Rusa unicolor*	RUN66	West Sumatra	H_46	MF177030	RUN	Naturalis	RMNH.MAM.1033.b	P. Kamminga	
*Rusa timorensis*	RTI2	Lesser Sunda Islands	H_31	MF177011		Berlin NHM	92305	F. Mayer	
*Rusa timorensis*	RTI3	Moluccas	H_1	MF176981		Berlin NHM	30840	F. Mayer	
*Rusa timorensis*	RTI6	Timor	H_2	MF176982	RTI	Stuttgart NHM	15878	S. Merker	
*Rusa timorensis*	RTI8	Java	H_3	MF176983		Stuttgart NHM	15892	S. Merker	
*Rusa timorensis*	RTI9	Java	H_4	MF176984		Stuttgart NHM	15897	S. Merker	
*Rusa timorensis*	RTI13	Java	H_1	MF176985		Stuttgart NHM	15891	S. Merker	
*Rusa timorensis*	RTI17	South Sulawesi	H_5	MF176986		Bonn NHM	58.109	R. Hutterer	
*Rusa timorensis*	RTI18	South Sulawesi	H_32	MF177012		Bonn NHM	58.75	R. Hutterer	
*Rusa timorensis*	RTI19	South Sulawesi	H_33	MF177013		Bonn NHM	58.105	R. Hutterer	
*Rusa timorensis*	RTI20	South Sulawesi	H_6	MF176987		Bonn NHM	58.112	R. Hutterer	
*Rusa timorensis*	RTI21	Timor	H_3	MF177014		Vienna NHM	231	F. Zachos	
*Rusa timorensis*	RTI22	Lesser Sunda Islands	H_34	MF177015		Vienna NHM	2049	F. Zachos	
*Rusa timorensis*	RTI27	Sulawesi	H_35	MF177016		Vienna NHM	7334	F. Zachos	
*Rusa timorensis*	RTI30	Java	H_7	MF176988		Dresden NHM	B730	C. Stefen	
*Rusa timorensis*	RTI31	Sulawesi	H_3	MF176989		Dresden NHM	B2686	C. Stefen	
*Rusa timorensis*	RTI32	Java	H_8	MF176990		Senckenberg Frankfurt	15462	I. Ruf	
*Rusa timorensis*	RTI33	Moluccas	H_9	MF176991		Senckenberg Frankfurt	5562	I. Ruf	
*Rusa timorensis*	RTI37	West Java	H_10	MF279247		Naturalis	RMNH.MAM.5679	P. Kamminga	
*Rusa timorensis*	RTI38	West Java	H_11	MF279248		Naturalis	RMNH.MAM.5680	P. Kamminga	
*Rusa timorensis*	RTI39	Timor	H_3	MF176992	RTI	Naturalis	RMNH.MAM.51419	P. Kamminga	
*Rusa timorensis*	RTI43	Timor	H_3	MF177017	RTI	Naturalis	RMNH.MAM.51417	P. Kamminga	
*Rusa timorensis*	RTI44	Bali	H_12	MF279249	RTI	Naturalis	RMNH.MAM.33828	P. Kamminga	
*Rusa timorensis*	RTI45	Bali	H_36	MF279252	RTI	Naturalis	RMNH.MAM.33827	P. Kamminga	

Samples are indicated according to the species assignment from the museum collections. mtDNA haplotype and genotypic cluster assignment are provided according to the results obtained.

**Table A2:** Microsatellite loci details and results

Locus	Allelic range	*N*_a_	*H*_E_	*H*_O_	*F*_IS_ (W&C)	Reference
Haut14	116–130	6	0.66	0.25	0.628	Kühn, Anastassiadis, and Pirchner (1996)
VH110	101–153	12	0.89	0.44	0.516	Talbot et al. (1996)
NVHRT48	71–120	12	0.8	0.31	0.615	Røed and Midthjell (1998)
BM757	167–199	12	0.92	0.5	0.424	Slate et al. (1998)
CSSM39	158–200	12	0.87	0.37	0.575	Slate et al. (1998)
CSSM41	120–146	11	0.85	0.37	0.566	Slate et al. (1998)
FSHB	171–206	13	0.92	0.5	0.426	Slate et al. (1998)
Roe09	167–199	12	0.9	0.63	0.31	Fickel and Reinsch (2000)
CSSM14	131–161	10	0.82	0.13	0.851	Kühn, Schröder, Pirchner, and Rottmann (2003)
T115	139–184	9	0.81	0.44	0.467	Meredith, Rodzen, Levine, and Banks (2005)
C143	154–174	4	0.71	0.19	0.741	Meredith et al. (2005)
C180	138–160	6	0.67	0.37	0.448	Meredith et al. (2005)
INRA6	103–141	10	0.78	0.37	0.529	Senn and Pemberton (2009)
Mu_4D	111–113	2	0.23	0	1.000	Schröder et al. (2016)
Mu_1_51	114–152	16	0.95	0.75	0.216	Schröder et al. (2016)
C183	119–133	5	0.65	0.25	0.62	Schröder et al. (2016)
Mu_1_25	98–110	6	0.78	0.37	0.529	Schröder et al. (2016)
Mu_1_550	139–173	14	0.94	0.37	0.608	Schröder et al. (2016)

Allelic range, number of alleles (*N*_a_), expected and observed heterozygosity (*H*_E_ and *H*_O_), and inbreeding coefficient (*F*_IS_) are given for each loci, as calculated for the 16 individuals genotyped.

## References

[ece33754-bib-0001] Bacon, C. D. , Henderson, A. J. , Mckenna, M. J. , Milroy, A. M. , & Simmons, M. P. (2013). Geographic and Taxonomic disparities in species diversity: Dispersal and diversification rates across Wallace's line. Evolution, 67, 2058–2071. https://doi.org/10.1111/evo.12084 23815659 10.1111/evo.12084

[ece33754-bib-0002] Bandelt, H. J. , Forster, P. , & Röhl, A. (1999). Median‐joining networks for inferring intraspecific phylogenies. Molecular Biology and Evolution, 16, 37–48. https://doi.org/10.1093/oxfordjournals.molbev.a026036 10331250 10.1093/oxfordjournals.molbev.a026036

[ece33754-bib-0003] Belkhir, K. , Borsa, P. , Chikki, L. , Raufaste, N. , & Bonhomme, F. (1996–2004) Genetix 4.05, logiciel sous Windows TM pour la genetique des populations. CNRS Umr 5000.

[ece33754-bib-0004] Bibi, F. (2013). A multi‐calibrated mitochondrial phylogeny of extant Bovidae (Artiodactyla, Ruminantia) and the importance of the fossil record to systematics. BMC Evolutionary Biology, 13, 166 https://doi.org/10.1186/1471-2148-13-166 23927069 10.1186/1471-2148-13-166 PMC3751017

[ece33754-bib-0005] Bird, M. I. , Taylor, D. , & Hunt, C. (2005). Palaeoenvironments of insular Southeast Asia during the Last Glacial Period: A savanna corridor in Sundaland? Quaternary Scientific Reviews, 24, 2228–2242. https://doi.org/10.1016/j.quascirev.2005.04.004

[ece33754-bib-0006] Chapuis, M. P. , & Estoup, A. (2007). Microsatellite null alleles and estimation of population differentiation. Molecular Biology and Evolution, 24, 621–631. 17150975 10.1093/molbev/msl191

[ece33754-bib-0007] Darriba, D. , Taboada, G. L. , Doallo, R. , & Posada, D. (2015). jModelTest 2: More models, new heuristics and high‐ performance computing. Nature Methods, 9, 6–9. 10.1038/nmeth.2109 PMC4594756 22847109

[ece33754-bib-0008] de Garine‐Wichatitsky, M. , de Meeus, T. , Chevillon, C. , Berthier, D. , Barre, N. , Thevenon, S. , & Maillard, J.‐C. (2009). Population genetic structure of wild and farmed rusa deer (*Cervus timorensis russa*) in New‐Caledonia inferred from polymorphic microsatellite loci. Genetica, 137, 313–323. https://doi.org/10.1007/s10709-009-9395-6 19680748 10.1007/s10709-009-9395-6

[ece33754-bib-0009] Demos, T. C. , Achmadi, A. S. , Giarla, T. C. , Handika, H. , Rowe, K. C. , & Esselstyn, J. A. (2016). Local endemism and within‐island diversification of shrews illustrate the importance of speciation in building Sundaland mammal diversity. Molecular Ecology, 25, 5158–5173. https://doi.org/10.1111/mec.13820 10.1111/mec.13820 27552382

[ece33754-bib-0010] Di Stefano, G. , & Petronio, C. (2002). Systematics and evolution of the Eurasian Plio‐Pleistocene tribe Cervini (Artiodactyla, Mammalia). Geologica Romana, 36, 311–334.

[ece33754-bib-0011] Drummond, A. J. , Suchard, M. A. , Xie, D. , & Rambaut, A. (2012). Bayesian phylogenetics with BEAUti and the BEAST 1.7. Molecular Biology and Evolution, 29, 1969–1973. https://doi.org/10.1093/molbev/mss075 22367748 10.1093/molbev/mss075 PMC3408070

[ece33754-bib-0012] Earl, D. A. , & von Holdt, B. M. (2012). STRUCTURE HARVESTER: A website and program for visualizing STRUCTURE output and implementing the Evanno method. Conservation Genetic Resources, 4, 359–361. https://doi.org/10.1007/s12686-011-9548-7

[ece33754-bib-0013] Emerson, B. C. , & Tate, M. L. (1993). Genetic analysis of evolutionary relationships among deer (Subfamily Cervinae). Journal of Heredity, 84, 266–273. https://doi.org/10.1093/oxfordjournals.jhered.a111338 8340615 10.1093/oxfordjournals.jhered.a111338

[ece33754-bib-0014] Escobedo‐Morales, L. A. , Mandujano, S. , Eguiarte, L. E. , Rodriguez‐Rodriguez, M. A. , & Maldonado, J. E. (2016). First phylogenetic analysis of Mesoamerican brocket deer *Mazama pandora* and *Mazama temama* (Cetartiodactyla: Cervidae) based on mitochondrial sequences: Implications on Neotropical deer evolution. Mammalian Biology, 81, 303–313. https://doi.org/10.1016/j.mambio.2016.02.003

[ece33754-bib-0015] Evanno, G. , Regnaut, S. , & Goudet, J. (2005). Detecting the number of clusters of individuals using the software STRUCTURE: A simulation study. Molecular Ecology, 14, 2611–2620. https://doi.org/10.1111/j.1365-294X.2005.02553.x 15969739 10.1111/j.1365-294X.2005.02553.x

[ece33754-bib-0016] Excoffier, L. , Laval, G. , & Schneider, S. (2005). Arlequin (version 3.0): An integrated software package for population genetics data analysis. Evolutionary Bioinformatics Online, 1, 47–50. PMC2658868 19325852

[ece33754-bib-0017] Fickel, J. , & Reinsch, A. (2000). Microsatellite markers for the European Roe deer (*Capreolus capreolus*). Molecular Ecology, 9, 993–1011. 10886662 10.1046/j.1365-294x.2000.00939-2.x

[ece33754-bib-0018] Fortes, G. G. , & Paijmans, J. L. A. (2015). Analysis of whole mitogenomes from ancient samples. Whole Genome Amplification Methods Protocols, 1347, 179–195. https://doi.org/10.1007/978-1-4939-2990-0 10.1007/978-1-4939-2990-0_13 26374318

[ece33754-bib-0019] Geist, V. (1998). Deer of the world. Their evolution, behaviour, and ecology. Mechanicsburg, PA: Stackpole Books.

[ece33754-bib-0020] Gorog, A. J. , Sinaga, M. H. , & Engstrom, M. D. (2004). Vicariance or dispersal? Historical biogeography of three Sunda shelf marine rodents (*Maxomys surifer*,* Leopoldamys sabanus* and *Maxomys whiteheadi*). Biological Journal of the Linnean Society, 81, 91–109. https://doi.org/10.1111/j.1095-8312.2004.00281.x

[ece33754-bib-0021] Groves, C. P. (1983). Pigs east of the Wallace Line. Journal de la Societe des Oceanistes, 39, 105–119. https://doi.org/10.3406/jso.1983.2792

[ece33754-bib-0022] Groves, C. P. , & Grubb, P. (2011). Ungulate Taxonomy. Baltimore, MD: The Jonhs Hopkins University Press.

[ece33754-bib-0023] Gupta, S. K. , Kumar, A. , Gaur, A. , & Hussain, S. A. (2015). Detection of 40 bp insertion ‐ deletion (INDEL) in mitochondrial control region among sambar (*Rusa unicolor*) populations in India. BMC Research Notes, 8, 1–7. 26483190 10.1186/s13104-015-1573-2 PMC4617744

[ece33754-bib-0024] Heany, L. R. (1991). A synopsis of climate and vegetational change in Southeast Asia. Climatic Change, 19, 53–61. https://doi.org/10.1007/BF00142213

[ece33754-bib-0025] Hedges, S. , Duckworth, J. W. , Timmins, R. J. , Semiadi, G. , & Dryden, G. (2015). *Rusa timorensis* . IUCN Red List Threatened Species, https://doi.org/10.2305/IUCN.UK.2015-2.RLTS.T41789A22156866.en

[ece33754-bib-0026] Heinsohn, T. (2003). Animal translocation: Long‐term human influences on the vertebrate zoogeography of Australasia (natural dispersal versus ethnophoresy). Australian Zoologist, 32, 351–376. https://doi.org/10.7882/AZ.2002.014

[ece33754-bib-0027] Katoh, K. , & Standley, D. M. (2013). MAFFT multiple sequence alignment software version 7: Improvements in performance and usability. Molecular Biology and Evolution, 30, 772–780. https://doi.org/10.1093/molbev/mst010 23329690 10.1093/molbev/mst010 PMC3603318

[ece33754-bib-0028] Kühn, R. , Anastassiadis, C. , & Pirchner, F. (1996). Transfer of bovine microsatellites to the cervine (*Cervus elaphus*). Animal Genetics, 27, 199–201. 8759122 10.1111/j.1365-2052.1996.tb00952.x

[ece33754-bib-0029] Kühn, R. , Schröder, W. , Pirchner, F. , & Rottmann, O. (2003). Genetic diversity, gene flow and drift in Bavarian red deer populations (*Cervus elaphus*). Conservation Genetics, 4, 157–166. https://doi.org/10.1023/A:1023394707884

[ece33754-bib-0030] Latinne, A. , Meynard, C. N. , Herbreteau, V. , Waengsothorn, S. , Morand, S. , & Michaux, J. R. (2015). Influence of past and future climate changes on the distribution of three Southeast Asian murine rodents. Journal of Biogeography, 42, 1714–1726. https://doi.org/10.1111/jbi.12528

[ece33754-bib-0031] Leonard, J. A. , den Tex, R.‐J. , Hawkins, M. T. R. , Munoz‐Fuentes, V. , Thorington, R. , & Maldonado, J. E. (2015). Phylogeography of vertebrates on the Sunda Shelf: A multi‐species comparison. Journal of Biogeography, 42, 871–879. https://doi.org/10.1111/jbi.12465

[ece33754-bib-0032] Leslie Jr, D. M. (2011). *Rusa unicolor* (Artiodactyla: Cervidae). Mammalian Species, 43, 1–30. https://doi.org/10.1644/871.1

[ece33754-bib-0033] Li, H. , & Durbin, R. (2009). Fast and accurate short read alignment with Burrows–Wheeler transform. Bioinformatics, 25, 1754–1760. https://doi.org/10.1093/bioinformatics/btp324 19451168 10.1093/bioinformatics/btp324 PMC2705234

[ece33754-bib-0034] Librado, P. , & Rozas, J. (2009). DnaSP v5: A software for comprehensive analysis of DNA polymorphism data. Bioinformatics, 25, 1451–1452. https://doi.org/10.1093/bioinformatics/btp187 19346325 10.1093/bioinformatics/btp187

[ece33754-bib-0035] Long, J. L. (2003). Introduced mammals of the world. Their history, distribution and influence. Clayton, VIC: CSIRO Publishing.

[ece33754-bib-0036] Maricic, T. , Whitten, M. , & Pääbo, S. (2010). Multiplexed DNA sequence capture of mitochondrial genomes using PCR products. PLoS ONE, 5, e14004 https://doi.org/10.1371/journal.pone.0014004 21103372 10.1371/journal.pone.0014004 PMC2982832

[ece33754-bib-0037] Martin, M. (2011). Cutadapt removes adapter sequences from high‐throughput sequencing reads. EMBnet Journal, 17, 10 https://doi.org/10.14806/ej.17.1.200

[ece33754-bib-0038] Martins, R. F. , Fickel, J. , Le, M. , van Nguyen, T. , Nguyen, H. M. , Timmins, R. , … Wilting, A. (2017). Phylogeography of red muntjacs reveals three distinct mitochondrial lineages. BMC Evolutionary Biology, 17, 34 https://doi.org/10.1186/s12862-017-0888-0 28122497 10.1186/s12862-017-0888-0 PMC5267393

[ece33754-bib-0039] Mattioli, S. (2011). Cervidae In Wilson D., & Mittermeier R. (Eds.), Handbook of the mammals of the World, Vol. 2 (pp. 350–443)., Hoofed mammals Barcelona, Spain: Lynx Edicions.

[ece33754-bib-0040] McKenna, A. , Hanna, M. , Banks, E. , Sivachenko, A. , Cibulskis, K. , Kernytsky, A. , … DePristo, M. A. (2010). The genome analysis Toolkit: A MapReduce framework for analyzing next‐generation DNA sequencing data. Genome Research, 20, 1297–1303. https://doi.org/10.1101/gr.107524.110 20644199 10.1101/gr.107524.110 PMC2928508

[ece33754-bib-0041] Meijaard, E. , & Groves, C. P. (2004). Morphometrical relationships between South‐east Asian deer (Cervidae, tribe Cervini): Evolutionary and biogeographic implications. Journal of the Zoological Society of London, 263, 179–196. https://doi.org/10.1017/S0952836904005011

[ece33754-bib-0042] Mercer, J. M. , & Roth, V. L. (2003). The effects of Cenozoic global change on squirrel phylogeny. Science, 299, 1568–1572. https://doi.org/10.1126/science.1079705 12595609 10.1126/science.1079705

[ece33754-bib-0043] Meredith, E. P. , Rodzen, J. A. , Levine, K. F. , & Banks, J. D. (2005). Characterization of an additional 14 microsatellite loci in California Elk (*Cervus elaphus*) for use in forensic and population applications. Conservation Genetics, 6, 151–153. https://doi.org/10.1007/s10592-004-7735-8

[ece33754-bib-0044] Mishra, S. , Gaillard, C. , Hertler, C. , Moigne, A. M. , & Simanjuntak, T. (2010). India and Java: Contrasting records, intimate connections. Quaternary International, 223–224, 265–270. https://doi.org/10.1016/j.quaint.2009.11.040

[ece33754-bib-0045] Muir, P. , Semiadi, G. , Asher, G. W. , Broad, T. E. , Tate, M. L. , & Barry, T. N. (1997). Sambar Deer (*Cervus unicolor*) × Red Deer (*C. elaphus*) interspecies hybrids. Journal of Heredity, 88, 366–372. https://doi.org/10.1093/oxfordjournals.jhered.a023120 9378911 10.1093/oxfordjournals.jhered.a023120

[ece33754-bib-0046] Patou, M.‐L. , Wilting, A. , Gaubert, P. , Esselstyn, J. A. , Cruaud, C. , Jennings, A. P. , … Veron, G. (2010). Evolutionary history of the Paradoxurus palm civets—A new model for Asian biogeography. Journal of Biogeography, 37, 2077–2097. https://doi.org/10.1111/j.1365-2699.2010.02364.x

[ece33754-bib-0047] Pitra, C. , Fickel, J. , Meijaard, E. , & Groves, C. (2004). Evolution and phylogeny of old world deer. Molecular Phylogenetics and Evolution, 33, 880–895. https://doi.org/10.1016/j.ympev.2004.07.013 15522810 10.1016/j.ympev.2004.07.013

[ece33754-bib-0048] Pritchard, J. K. , Stephens, M. , & Donnelly, P. (2000). Inference of population structure using multilocus genotype data. Genetics, 155, 945–959. 10835412 10.1093/genetics/155.2.945 PMC1461096

[ece33754-bib-0049] Røed, K. H. , & Midthjell, L. (1998). Microsatellites in reindeer, *Rangifer tarandus*, and their use in other cervids. Molecular Ecology, 7, 1771–1788. 9859205 10.1046/j.1365-294x.1998.00514.x

[ece33754-bib-0050] Ronquist, F. , & Huelsenbeck, J. P. (2003). MrBayes 3: Bayesian phylogenetic inference under mixed models. Bioinformatics, 19, 1572–1574. https://doi.org/10.1093/bioinformatics/btg180 12912839 10.1093/bioinformatics/btg180

[ece33754-bib-0051] Sathiamurthy, E. , & Voris, K. H. (2006). Maps of Holocene Sea Level Transgression and Submerged Lakes on the Sunda Shelf. The Natural History Journal of Chulalongkorn University, Suppl., 2, 1–43.

[ece33754-bib-0052] Schröder, O. , Lieckfeldt, D. , Lutz, W. , Rudloff, C. , Frölich, K. , & Ludwig, A. (2016). Limited hybridisation between domestic and the European mouflon in Western Germany. European Journal of Wildlife Research, 62, 307–314. https://doi.org/10.1007/s10344-016-1003-3

[ece33754-bib-0053] Senn, H. V. , & Pemberton, J. M. (2009). Variable extent of hybridization between invasive sika (*Cervus nippon*) and native red deer (*C. elaphus*) in a small geographical area. Molecular Ecology, 18, 862–876. https://doi.org/10.1111/j.1365-294X.2008.04051.x 19175500 10.1111/j.1365-294X.2008.04051.x

[ece33754-bib-0054] Sheldon, F. H. , Lim, H. C. , & Moyle, R. G. (2015). Return to the Malay Archipelago: The biogeography of Sundaic rainforest birds. Journal of Ornithology, 156, 90–113.

[ece33754-bib-0055] Silvestro, D. , & Michalak, I. (2012). RaxmlGUI: A graphical front‐end for RAxML. Organisms Diversity & Evolution, 12, 335–337. https://doi.org/10.1007/s13127-011-0056-0

[ece33754-bib-0056] Slate, J. , Coltman, D. W. , Goodman, S. J. , MacLean, I. , Pemberton, J. M. , & Williams, J. L. (1998). Bovine microsatellite loci are highly conserved in red deer (*Cervus elaphus*), sika deer (*Cervus nippon*) and Soay sheep (*Ovis aries*). Animal Genetics, 29, 307–315. https://doi.org/10.1046/j.1365-2052.1998.00347.x 9745670 10.1046/j.1365-2052.1998.00347.x

[ece33754-bib-0057] Smith, S. L. , Carden, R. F. , Coad, B. , Birkitt, T. , & Pemberton, J. M. (2014). A survey of the hybridisation status of Cervus deer species on the island of Ireland. Conservation Genetics, 15, 823–835. https://doi.org/10.1007/s10592-014-0582-3

[ece33754-bib-0058] Sun, X. , Li, X. , Luo, Y. , & Chen, X. (2000). The vegetation and climate at the last glaciation on the emerged continental shelf of the South China Sea. Palaeogeography, Palaeoclimatology and Palaeoecology, 160, 301–316. https://doi.org/10.1016/S0031-0182(00)00078-X

[ece33754-bib-0501] Talbot, J. , Haigh, J. , & Plante, Y. (1996). A parentage evaluation test in North American Elk (Wapiti) using microsatellites from ovine and bovine origin. Animal Genetics, 27, 117–119. https://doi.org/10.1111/j.1365-2052.1996.tb00480.x 8856904 10.1111/j.1365-2052.1996.tb00480.x

[ece33754-bib-0059] Timmins, R.J. , Kawanishi, K. , Giman, B. , Lynam, A. , Chan, B. , Steinmetz, R. , … Samba Kumar, N. (2015) Rusa unicolor. IUCN Red List Threatened Species, e.T41790A85628124.

[ece33754-bib-0060] van Bemmel, A. C. V. (1949). Revision of the Rusine Deer. Treubia, 20, 191–262.

[ece33754-bib-0061] Van Den Bergh, G. D. , De Vos, J. , & Sondaar, P. Y. (2001). The Late Quaternary palaeogeography of mammal evolution in the Indonesian Archipelago. Palaeogeography, Palaeoclimatology and Palaeoecology, 171, 385–408. https://doi.org/10.1016/S0031-0182(01)00255-3

[ece33754-bib-0062] Veron, G. , Willsch, M. , Dacosta, V. , Patou, M.‐L. , Seymour, A. , Bonillo, C. , … Wilting, A. (2014). The distribution of the Malay civet *Viverra tangalunga* (Carnivora: Viverridae) across Southeast Asia: Natural or human‐mediated dispersal? Zoological Journal of the Linnean Society, 170, 917–932. https://doi.org/10.1111/zoj.12110

[ece33754-bib-0063] Voris, H. K. (2000). Maps of Pleistocene sea levels in Southeast Asia: Shorelines, river systems and time durations. Journal of Biogeography, 27, 1153–1167. https://doi.org/10.1046/j.1365-2699.2000.00489.x

[ece33754-bib-0064] Webley, L. S. , Zenger, K. R. , English, A. W. , & Cooper, D. W. (2004). Low levels of genetic variation within introduced Javan rusa deer (*Cervus timorensis russa*) in Australia. European Journal of Wildlife Research, 50, 137–140.

[ece33754-bib-0065] Zazo, C. (1999). Interglacial sea levels. Quaternary International, 55, 101–113. https://doi.org/10.1016/S1040-6182(98)00031-7

